# Inflammatory–Hematological Profiles in Nasopharyngeal Carcinoma and Suspicious Adenoid Hypertrophy: An Exploratory Single-Center Study

**DOI:** 10.3390/diagnostics16152469

**Published:** 2026-08-05

**Authors:** Darius Radu Roman, Carmen Delia Nistor-Cseppento, Dana Carmen Zaha, Timea Claudia Ghitea, Alexia Manole, Dana Zdremtan, Alexandru Chioreanu, Daniela Florina Trifan, Palade Octavian Dragoș, Felicia Manole

**Affiliations:** 1 Doctoral School of Biomedical Sciences, Faculty of Medicine and Pharmacy, University of Oradea, 410087 Oradea, Romania; roman.dariusradu@student.uoradea.ro; 2 Department of Psycho-Neuroscience and Recovery, Faculty of Medicine and Pharmacy, University of Oradea, 410073 Oradea, Romania; dcseppento@uoradea.ro; 3 Department of Preclinical Disciplines, Faculty of Medicine and Pharmacy, University of Oradea, 410073 Oradea, Romania; danaczaha@gmail.com; 4 Department of Pharmacy, Faculty of Medicine and Pharmacy, University of Oradea, 410073 Oradea, Romania; 5 Faculty of Medicine and Pharmacy, University of Oradea, 410068 Oradea, Romania; manole.alexia@student.uoradea.ro; 6 Department of Biochemistry and ENT, “Vasile Goldis” Western University, B-dul Revolutiei Nr. 96, 310025 Arad, Romania; zdremtan.dana@uvvg.ro (D.Z.); chioreanu.alexandru@uvvg.ro (A.C.); 7 Medicine Department, Faculty of Medicine and Pharmacy, University of Oradea, 410068 Oradea, Romania; daniela.trifan@csud.uoradea.ro (D.F.T.); fmanole@uoradea.ro (F.M.); 8 St. Spiridon County Emergency Hospital Iași, B-dul Independenței 1, 700111 Iași, Romania; octavian.palade@umfiasi.ro

**Keywords:** nasopharyngeal carcinoma, adenoid hypertrophy, C-reactive protein, inflammatory biomarkers, neutrophil-to-lymphocyte ratio, platelet-to-lymphocyte ratio, systemic immune-inflammation index, differential diagnosis, Firth logistic regression

## Abstract

**Background**: Differentiating nasopharyngeal carcinoma (NPC) from benign nasopharyngeal lesions may be challenging because clinical and endoscopic findings can overlap. This study compared routine inflammatory and hematological biomarkers between patients with histopathologically confirmed NPC and patients with suspicious but histopathologically benign adenoid hypertrophy (AH). **Methods**: This retrospective single-center study included 72 adults evaluated between January 2024 and January 2026: 36 patients with NPC and 36 with AH. Routine hematological and inflammatory variables were compared between groups. After inconsistencies were identified in the originally derived indices, the neutrophil-to-lymphocyte ratio (NLR), platelet-to-lymphocyte ratio (PLR), and systemic immune-inflammation index (SII) were recalculated using the available absolute blood-cell-count variables. Receiver operating characteristic analyses and exploratory Firth penalized logistic regression were performed, with histopathologically confirmed NPC coded as the positive outcome. **Results**: Patients with NPC were younger than patients with AH (38.14 ± 7.64 vs. 56.06 ± 8.29 years; *p* < 0.001). CRP, ESR, and leukocyte count were significantly higher in the NPC group. PLR was significantly higher in the AH group, whereas NLR and SII did not differ significantly between groups. CRP demonstrated apparent complete discrimination between the two selected diagnostic groups (AUC 1.000), while ESR yielded an AUC of 0.948. In the Firth penalized logistic regression model adjusted for age, sex, and smoking status, each 10 mg/L increase in CRP was associated with higher odds of NPC (adjusted OR 3.16, 95% CI 1.68–16.72; *p* < 0.001). The addition of CRP increased the model AUC from 0.950 to 1.000. **Conclusions**: Routine inflammatory markers showed different cross-sectional distributions between patients with node-positive NPC and patients with suspicious benign adenoid hypertrophy. CRP provided incremental discriminatory information in this selected cohort but should not be interpreted as a validated stand-alone diagnostic marker. The findings require prospective external validation in a larger and clinically representative population.

## 1. Introduction

Nasopharyngeal carcinoma (NPC) represents a distinct malignant epithelial tumor arising from the nasopharyngeal mucosa, characterized by unique epidemiological, biological, and clinical features compared with other head and neck malignancies. Although relatively uncommon in European populations, NPC remains an important oncological challenge due to its aggressive loco-regional behavior, tendency for early lymphatic dissemination, and frequently delayed diagnosis. The disease exhibits marked geographical variability, with higher incidence rates reported in Southeast Asia, North Africa, and certain Mediterranean regions, suggesting the involvement of complex genetic, environmental, infectious, and immunological mechanisms in its pathogenesis [1,2,3].

The etiopathogenesis of nasopharyngeal carcinoma is multifactorial and incompletely understood. Chronic inflammation, Epstein–Barr virus (EBV) infection, environmental carcinogens, dietary factors, tobacco exposure, and immune dysregulation have all been implicated in malignant transformation and tumor progression. Increasing evidence suggests that systemic inflammatory activation plays a major role in tumor initiation, angiogenesis, tissue invasion, immune escape, and metastatic dissemination. Inflammatory mediators may influence both the tumor microenvironment and the systemic host response, contributing to disease heterogeneity and variability in clinical outcomes [4,5,6].

One of the major clinical challenges in nasopharyngeal pathology is the differential diagnosis between malignant nasopharyngeal lesions and benign inflammatory conditions, particularly adenoid hypertrophy and chronic lymphoid hyperplasia [7,8]. Patients with benign adenoidal disease may present with symptoms and endoscopic findings partially overlapping with nasopharyngeal carcinoma, including nasal obstruction, otologic symptoms, mucosal hypertrophy, cervical lymphadenopathy, and inflammatory secretions. This overlap may delay diagnosis or complicate clinical decision-making, especially in patients presenting with suspicious inflammatory or proliferative lesions [9,10,11,12,13].

Routine inflammatory and hematological biomarkers have gained increasing interest as accessible tools capable of reflecting systemic inflammatory activity and tumor-associated immune alterations. Parameters such as leukocyte count, neutrophils, lymphocytes, C-reactive protein (CRP), erythrocyte sedimentation rate (ESR), and derived inflammatory indices including neutrophil-to-lymphocyte ratio (NLR), platelet-to-lymphocyte ratio (PLR), and systemic immune-inflammation index (SII) have been investigated in various oncological diseases as potential markers of disease severity, prognosis, and treatment response [14,15,16].

These biomarkers are attractive because they are inexpensive, widely available, reproducible, and easily integrated into routine clinical evaluation. However, their interpretation in nasopharyngeal pathology remains challenging. While elevated inflammatory markers are often associated with malignant disease, benign inflammatory conditions may also induce intense systemic immune activation, potentially mimicking oncological inflammatory profiles. Consequently, the biological distinction between malignant and benign nasopharyngeal inflammatory phenotypes remains insufficiently explored [17,18].

Recent oncological studies suggest that composite inflammatory indices may reflect complex interactions between innate immunity, lymphoid activation, platelet-mediated inflammation, and tumor-associated immune dysregulation. Nevertheless, most available studies have focused primarily on prognosis in confirmed NPC, while limited data are available regarding the comparative inflammatory behavior between nasopharyngeal carcinoma and benign adenoidal pathology presenting with clinical suspicion of malignancy [19,20].

In this context, the present study aimed to compare routine inflammatory and hematological biomarkers between patients with histopathologically confirmed nasopharyngeal carcinoma and patients with histopathologically benign adenoid hypertrophy evaluated because malignancy was clinically suspected.

The primary objective was to determine whether CRP, ESR, leukocyte count, NLR, PLR, and SII differed between the two diagnostic groups. The secondary objective was to explore the discriminatory performance of these biomarkers and to assess whether CRP provided incremental information beyond basic demographic and clinical variables. All analyses were considered exploratory because of the retrospective design, selected comparator group, limited sample size, and absence of external validation.

## 2. Materials and Methods

### 2.1. Study Design and Population

This retrospective single-center observational study included 72 adult patients evaluated for suspicious nasopharyngeal lesions in the Otorhinolaryngology Department between January 2024 and January 2026. Patients underwent clinical examination, nasopharyngeal endoscopy, and, where clinically indicated, imaging and histopathological evaluation.

The final analytical cohort comprised 36 patients with histopathologically confirmed nasopharyngeal carcinoma (NPC) and 36 patients with histopathologically confirmed benign adenoid hypertrophy (AH). The AH group consisted of patients who underwent diagnostic assessment because their clinical, endoscopic, and/or imaging findings raised suspicion of malignancy. Therefore, this comparator group should not be considered representative of uncomplicated adenoid hypertrophy in the general population.

All consecutive eligible patients evaluated during the study period were screened for inclusion. After applying the predefined inclusion and exclusion criteria, the final analytical cohort consisted of 36 patients with histopathologically confirmed NPC and 36 patients with histopathologically confirmed benign adenoid hypertrophy. No matching or random sampling was performed. The equal group sizes resulted from the number of eligible patients available during the study period rather than from intentional balancing. No matching or additional sampling procedure was applied. Eligible patients were required to have pretreatment hematological and inflammatory laboratory measurements available before the initiation of oncological therapy. All patients in the NPC group had laterocervical lymph-node involvement at diagnosis, indicating that the malignant cohort represented a selected node-positive NPC population.

Epstein–Barr virus DNA, EBV serological parameters, and detailed TNM staging information were not systematically available in the retrospective database and were therefore not included in the analysis.

The inclusion criteria were:

- Age ≥ 18 years;
- Histopathological confirmation of NPC or benign adenoid hypertrophy;
- Availability of pretreatment hematological and inflammatory laboratory data required for the planned analyses;
- Completed nasopharyngeal endoscopic evaluation.

The exclusion criteria were:

- Acute systemic infection documented at the time of laboratory assessment;
- Known autoimmune or active chronic inflammatory disease with a potential influence on inflammatory biomarkers;
- Hematological malignancy;
- Previous oncological treatment before laboratory assessment;
- Absence of histopathological confirmation;
- Insufficient or unverifiable data for the principal analyses.

**Figure 1:**
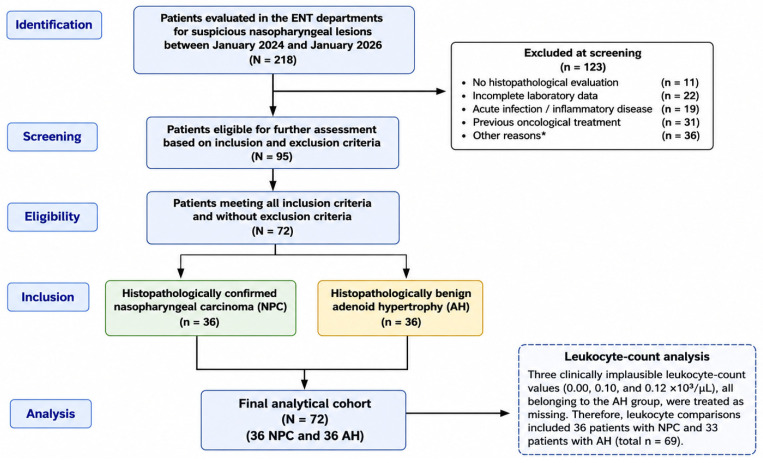
STROBE flow diagram illustrating patient screening, eligibility assessment, exclusions, and inclusion in the final analytical cohort. All consecutive eligible adult patients evaluated for suspicious nasopharyngeal lesions between January 2024 and January 2026 were screened. After application of the predefined inclusion and exclusion criteria, the final cohort consisted of 36 patients with histopathologically confirmed nasopharyngeal carcinoma (NPC) and 36 patients with histopathologically confirmed benign adenoid hypertrophy (AH). No matching or random sampling procedure was performed. * Other reasons included administrative exclusions, duplicate records, insufficient clinical documentation, patient withdrawal, or other protocol-defined reasons precluding eligibility assessment.

The STROBE flow diagram illustrating patient screening, eligibility assessment, exclusions, and inclusion in the final analytical cohort is presented in Figure 1.

### 2.2. Clinical and Endoscopic Evaluation

All patients underwent a complete otorhinolaryngological examination, including endoscopic evaluation of the nasopharynx. The endoscopic examination documented the visible characteristics of the nasopharyngeal lesion, including mucosal hypertrophy, proliferative or infiltrative appearance, surface irregularity, ulceration or necrosis, inflammatory secretions, tubal obstruction, and the visible local extent of the lesion.

Laterocervical lymph-node involvement was recorded separately as a clinical characteristic and was not considered an endoscopic variable.

Representative endoscopic images from patients with NPC and AH were included for illustrative purposes only. The present study did not perform a standardized quantitative comparison of individual endoscopic findings and did not evaluate whether endoscopy provided incremental diagnostic performance beyond the laboratory biomarkers.

### 2.3. Laboratory Parameters

Routine pretreatment laboratory data were extracted retrospectively from the hospital medical records. Only measurements obtained before the initiation of oncological treatment were considered. The analyzed variables included hematological, inflammatory, biochemical, and coagulation parameters.

Hematological variables included leukocyte count, erythrocyte count, hemoglobin, hematocrit, platelet count, absolute neutrophil count, absolute lymphocyte count, absolute monocyte count, absolute eosinophil count, and absolute basophil count.

The inflammatory biomarkers included C-reactive protein (CRP), erythrocyte sedimentation rate (ESR), neutrophil-to-lymphocyte ratio (NLR), platelet-to-lymphocyte ratio (PLR), and systemic immune-inflammation index (SII).

Absolute neutrophil count, absolute lymphocyte count, and platelet count were obtained from the same pretreatment complete blood count. Cell counts were expressed in ×10^3^/µL. The derived inflammatory indices were recalculated as follows:

$$NLR=\frac{Absolute\;neutrophil\;count}{Absolute\;lymphocyte\;count}$$

$$PLR=\frac{Platelet\;count}{Absolute\;lymphocyte\;count}$$

$$SII=\frac{Platelet\;count\times Absolute\;neutrophil\;count}{Absolute\;lymphocyte\;count}$$

NLR and PLR are dimensionless ratios, whereas SII is expressed in ×10^3^/µL. Percentage differential counts were not used to calculate the derived indices.

The biochemical variables included glucose, urea, creatinine, total cholesterol, triglycerides, aspartate aminotransferase (AST), alanine aminotransferase (ALT), gamma-glutamyl transferase (GGT), and uric acid.

Coagulation parameters included the international normalized ratio (INR), prothrombin index (IQ), and activated partial thromboplastin time (APTT).

### 2.4. Statistical Analysis

A patient-level data audit was performed before the statistical analyses. The originally calculated NLR and SII variables were discarded because they combined neutrophil percentages with absolute lymphocyte counts. NLR, PLR, and SII were recalculated using the available absolute neutrophil, absolute lymphocyte, and platelet counts from the same pretreatment complete blood count.

Three leukocyte-count values of 0.00, 0.10, and 0.12 × 10^3^/µL were considered clinically implausible and were treated as missing in leukocyte-dependent analyses, resulting in 69 evaluable patients for leukocyte count. No missing laboratory value was imputed.

Categorical variables were summarized as counts and percentages. Age was reported as mean ± standard deviation and compared between groups using Welch’s *t*-test, with the mean difference and 95% confidence interval. Laboratory variables were reported as median and interquartile range and compared using the Mann–Whitney U test because their distributions were non-normal. Categorical variables were compared using Fisher’s exact test.

Receiver operating characteristic (ROC) analyses were performed with histopathologically confirmed NPC coded as the positive outcome. Higher values were considered indicative of NPC for CRP, ESR, leukocyte count, and GGT. Because lower values of NLR, PLR, and SII were associated with NPC in the analyzed dataset, their ROC direction was specified as “lower values indicating NPC”.

Areas under the ROC curve were reported with 95% confidence intervals and *p*-values. Exploratory thresholds were identified by maximizing the Youden index. Sensitivity and specificity were reported with exact 95% Clopper–Pearson confidence intervals.

Multivariable analysis was performed using Firth’s penalized binary logistic regression, with histopathological diagnosis coded as 1 for NPC and 0 for AH. The baseline clinical model included age, sex, and smoking status. The expanded model included the same variables together with CRP, expressed per 10 mg/L increase.

Firth’s penalized likelihood method was selected because CRP completely separated the two diagnostic groups and conventional logistic regression would have produced unstable or infinite coefficient estimates. Adjusted odds ratios were reported with profile penalized-likelihood 95% confidence intervals and penalized likelihood-ratio *p*-values.

Model discrimination was evaluated using the area under the ROC curve. The baseline and expanded models were compared using the absolute change in AUC, the DeLong test, and the penalized likelihood-ratio test. Sensitivity and specificity were calculated at a predicted-probability threshold of 0.50. Prediction error and calibration were evaluated using the Brier score, calibration intercept, and calibration slope. Internal validation was performed using 1000 stratified bootstrap resamples.

Laterocervical lymph-node involvement was not included in the regression models because it completely separated the diagnostic groups, being present in all NPC patients and absent in the AH group. EBV-related parameters and detailed TNM stage were not available in the retrospective database and were therefore not included in the adjusted analyses.

All tests were two-sided, and *p*-values < 0.05 were considered statistically significant. Because of the retrospective design, limited sample size, selected comparator group, and absence of external validation, all analyses were considered exploratory.

Statistical analyses were performed using IBM SPSS Statistics version 30 and Python version 3.13. NumPy version 2.3.5, SciPy version 1.17.0, and scikit-learn version 1.8.0 were used for data audit, ROC calculations, penalized regression, model-performance evaluation, and bootstrap validation. Canva Pro (Canva Pty Ltd., Sydney, Australia; web-based graphic design platform, accessed April 2026) was used only for final graphical layout and was not used for statistical calculations.

### 2.5. Ethical Considerations

The study was conducted in accordance with the ethical principles outlined in the Declaration of Helsinki and respected institutional requirements regarding confidentiality and protection of personal medical data.

Ethical approval for the study entitled “Managementul diagnosticului, implicații clinice și etiopatogenice în cancerul de rinofaringe” was granted by the Research Ethics Subcommittee of the University of Oradea under approval number 80/31 March 2026.

Data extraction began in April 2026, and statistical analysis began in April 2026. Both procedures were conducted after institutional ethical approval. All patient-level data were anonymized before analysis.

## 3. Results

### 3.1. Demographic and Biological Characteristics of the Study Population

The study included 72 patients. The mean age was 47.10 ± 12.00 years, ranging from 24 to 71 years. After three clinically implausible leukocyte-count values (0.00, 0.10, and 0.12 × 10^3^/µL) were treated as missing, leukocyte count was available for 69 patients, with an overall mean value of 13.50 ± 5.76 × 10^3^/µL. The demographic and laboratory characteristics of the study cohort are summarized in Table 1. Group-specific comparisons are presented in Table 2. CRP and ESR showed wide distributions, with mean values of 42.71 ± 42.40 mg/L and 34.19 ± 45.58 mm/h, respectively. The recalculated inflammatory indices had mean values of 1.80 ± 0.72 for NLR, 78.06 ± 23.90 for PLR, and 454.36 ± 168.62 × 10^3^/µL for SII. Complete coefficient output for the baseline Firth penalized logistic regression model is presented in Table S1.

Platelet counts demonstrated moderate variability (mean 263.10 ± 45.26 × 10^3^/µL), while inflammatory markers showed marked dispersion across the cohort. CRP values ranged from minimal elevations to markedly increased levels, reaching a maximum value of 105 mg/L.

Biochemical analyses revealed variability in hepatic and metabolic markers, including AST, ALT, GGT, glucose, and uric acid levels, suggesting potential systemic metabolic involvement associated with tumor-related inflammatory processes.

The cohort showed substantial variability in routine inflammatory and hematological measurements. CRP and ESR displayed wide distributions, while leukocyte count was available for 69 patients after three clinically implausible values were treated as missing. Descriptive characteristics of the study cohort are presented in Table 1. Overall, the biological profile of the cohort was characterized by predominantly inflammatory–hematological alterations, supporting the hypothesis that routine laboratory biomarkers may reflect disease burden and loco-regional extension in patients with nasopharyngeal carcinoma.

### 3.2. Comparative Inflammatory Profiles Between Nasopharyngeal Carcinoma and Adenoid Hypertrophy

Patients were divided into two study groups according to the histopathological and clinical diagnosis:

- Group 1: patients diagnosed with nasopharyngeal carcinoma (NPC);
- Group 2: patients presenting adenoid hypertrophy/vegetations with clinical suspicion of malignancy.

After the three clinically implausible leukocyte-count values were treated as missing, leukocyte count was available for 69 patients, including 36 patients with NPC and 33 patients with AH. The overall mean leukocyte count was 13.50 ± 5.76 × 10^3^/µL. Leukocyte values were significantly higher in the NPC group than in the AH group, with median values of 17.43 × 10^3^/µL (IQR 15.06–20.15) and 8.50 × 10^3^/µL (IQR 8.16–9.50), respectively (Mann–Whitney U test, *p* < 0.001). Comparative analysis also demonstrated significantly higher CRP and ESR values in the NPC group than in the AH group.

Interestingly, derived inflammatory biomarkers including NLR, PLR, and SII demonstrated paradoxically higher values in the adenoid hypertrophy group compared with the NPC group.

Table S2 describes the complete coefficient output for the CRP-expanded Firth penalized logistic regression model.

However, overlap between the two groups was observed in several routine biomarkers, indicating that benign inflammatory adenoidal disease may clinically and biologically mimic nasopharyngeal carcinoma in selected cases.

Individual variation in inflammatory-marker values was observed within the NPC group; however, the study was not designed to define or validate a distinct inflammatory phenotype.

Representative endoscopic images from patients with NPC and AH are presented for illustrative purposes only. No formal comparative analysis of endoscopic characteristics was performed.

**Figure 2:**
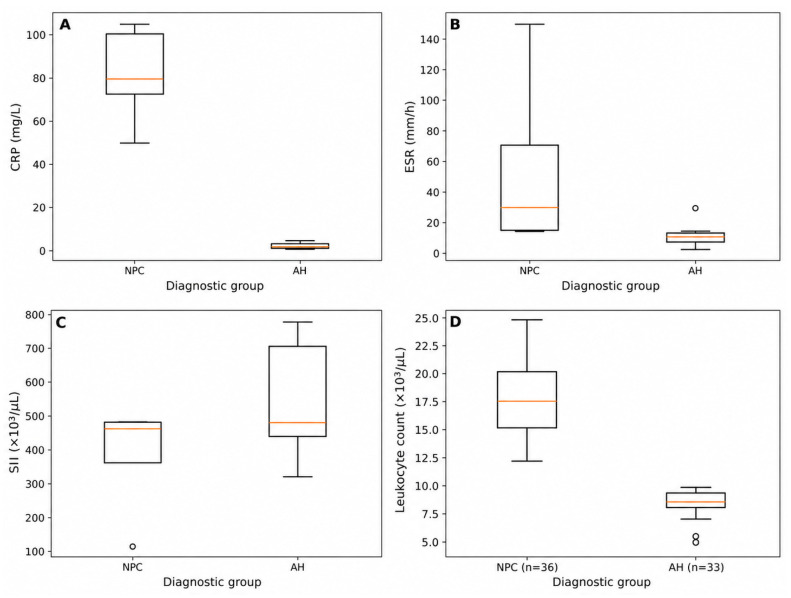
Comparative distributions of selected inflammatory biomarkers between patients with nasopharyngeal carcinoma and patients with suspicious histopathologically benign adenoid hypertrophy. (**A**) C-reactive protein concentrations; (**B**) erythrocyte sedimentation rate; (**C**) recalculated systemic immune-inflammation index; and (**D**) leukocyte count. Three clinically implausible leukocyte-count values (0.00, 0.10, and 0.12 × 10^3^/µL) were treated as missing; therefore, the leukocyte-count analysis included 69 evaluable patients. Boxplots display the median, interquartile range, whiskers, and outlying observations. CRP, C-reactive protein; ESR, erythrocyte sedimentation rate; SII, systemic immune-inflammation index.

Overall, the results support the potential role of routine inflammatory and hematological biomarkers as complementary low-cost tools in the differential diagnostic assessment between nasopharyngeal carcinoma and benign adenoidal inflammatory pathology (Table 2 and Figure 2).

### 3.3. Correlation Analysis of Inflammatory and Hematological Biomarkers

Correlation analysis demonstrated strong positive associations among the classical inflammatory markers in the pooled cohort. CRP correlated positively with ESR (Spearman’s ρ = 0.795, *p* < 0.001) and leukocyte count (ρ = 0.883, *p* < 0.001), while ESR was also strongly correlated with leukocyte count (ρ = 0.875, *p* < 0.001). Among the recalculated derived indices, NLR showed a strong positive correlation with SII (ρ = 0.811, *p* < 0.001), whereas its correlation with PLR was weaker (ρ = 0.372, *p* = 0.001). PLR was moderately correlated with SII (ρ = 0.554, *p* < 0.001). CRP was inversely correlated with NLR (ρ = −0.320, *p* = 0.006), PLR (ρ = −0.837, *p* < 0.001), and SII (ρ = −0.470, *p* < 0.001). ESR was inversely correlated with NLR (ρ = −0.258, *p* = 0.029) and PLR (ρ = −0.599, *p* < 0.001), but not significantly with SII (ρ = −0.163, *p* = 0.172). Leukocyte count was inversely correlated with PLR (ρ = −0.797, *p* < 0.001), whereas its associations with NLR and SII were not statistically significant. Because the correlations were calculated in the pooled cohort, they may partly reflect between-group differences and should be interpreted descriptively rather than mechanistically.

**Figure 3:**
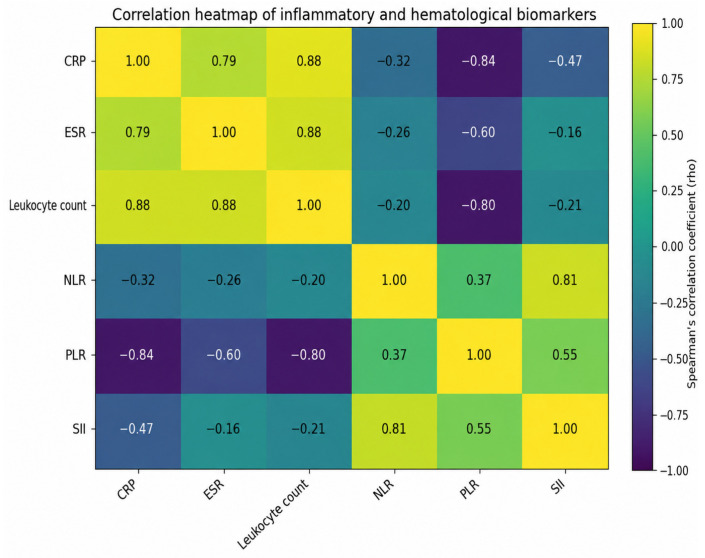
Spearman correlation heatmap of inflammatory and hematological biomarkers in the pooled study cohort. NLR, PLR, and SII were recalculated using absolute neutrophil and lymphocyte counts obtained from the same pretreatment complete blood count. Three clinically implausible leukocyte-count values were treated as missing; therefore, correlations involving leukocyte count were based on 69 evaluable patients, whereas the remaining correlations included 72 patients. Correlations were calculated using pairwise complete observations. CRP, C-reactive protein; ESR, erythrocyte sedimentation rate; NLR, neutrophil-to-lymphocyte ratio; PLR, platelet-to-lymphocyte ratio; SII, systemic immune-inflammation index.

These findings are descriptive and should be interpreted cautiously given the selected study population (Figure 3).

Apparent and bootstrap-corrected performance of the baseline and CRP-expanded Firth logistic regression models is presented in Table S3, and the complete statistical comparison of the baseline clinical and CRP-expanded Firth logistic regression modelsin Table S4.

### 3.4. Diagnostic Performance of Routine Inflammatory Biomarkers

Receiver operating characteristic (ROC) curve analysis was performed to explore the diagnostic performance of routinely available inflammatory and hematological biomarkers for differentiating histopathologically confirmed nasopharyngeal carcinoma (NPC) from suspicious but histopathologically benign adenoid hypertrophy (AH).

Leukocyte count demonstrated apparent complete discrimination between the two selected diagnostic groups, with an AUC of 1.000. The exploratory threshold maximizing the Youden index was >10.97 × 10^3^/µL, yielding a sensitivity of 100.0% (95% CI 90.3–100.0%) and a specificity of 100.0% (95% CI 89.4–100.0%). These estimates should be interpreted cautiously because they were obtained in a small, selected cohort and require external validation (Table 3).

CRP and leukocyte count completely separated the two diagnostic groups in the analyzed dataset, each yielding an apparent AUC of 1.000. ESR also demonstrated excellent discriminatory performance (AUC = 0.948, 95% CI 0.889–0.988, *p* < 0.001). Among the derived inflammatory indices, only PLR showed statistically significant discrimination (AUC = 0.833, 95% CI 0.725–0.915, *p* < 0.001), with lower values observed in patients with NPC. Neither NLR (AUC = 0.590, *p* = 0.188) nor SII (AUC = 0.604, *p* = 0.128) demonstrated significant discriminatory ability. Likewise, GGT showed poor diagnostic performance (AUC = 0.406, *p* = 0.171).

The distribution of the derived inflammatory indices differed from that of the classical inflammatory biomarkers. In particular, PLR was significantly lower in NPC than in AH, whereas NLR and SII did not demonstrate significant discriminatory performance. These findings should be considered exploratory and may reflect the selected comparator group, age imbalance, residual confounding, or measurement-related factors.

**Figure 4:**
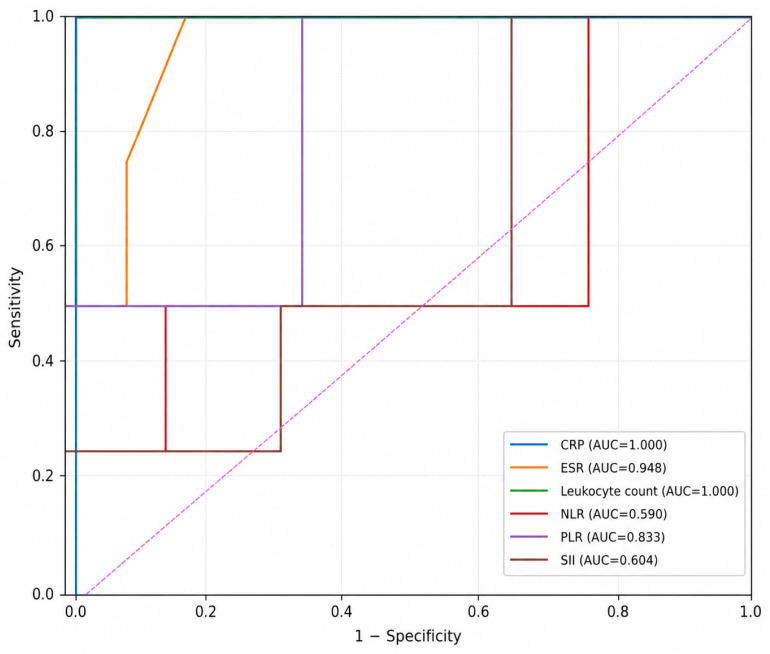
Receiver operating characteristic curves for routine inflammatory and hematological biomarkers. NPC was coded as the positive outcome. Higher CRP, ESR, and leukocyte-count values and lower PLR values were associated with NPC in the analyzed cohort. The discriminatory performance of NLR, SII, and GGT was not statistically significant. Because the study included a selected case–control cohort without external validation, the reported ROC estimates should be interpreted as exploratory.

The present study did not evaluate whether these laboratory biomarkers provide incremental diagnostic value beyond routine clinical examination, endoscopic assessment, or imaging. Therefore, the observed ROC results should be interpreted as hypothesis-generating rather than as evidence supporting the independent clinical use of these biomarkers (Figure 4).

### 3.5. Exploratory Adjusted Regression Analysis

Firth penalized logistic regression was used because CRP completely separated the two diagnostic groups. The baseline model included age, sex, and smoking status, and the expanded model additionally included CRP per 10 mg/L increase.

In the baseline model, increasing age was associated with lower odds of NPC in this selected cohort (adjusted OR 0.75, 95% CI 0.62–0.84; *p* < 0.001). Sex and smoking status were not statistically significant.

After CRP was added, each 10 mg/L increase in CRP was associated with higher odds of NPC (adjusted OR 3.16, profile penalized-likelihood 95% CI 1.68–16.72; *p* < 0.001).

The apparent AUC increased from 0.950 to 1.000. The expanded model had an ap-parent Brier score of 0.0024. These results reflect the complete separation of CRP values and the selected retrospective cohort.

Model-performance measures are summarized in Table 4. Internal bootstrap estimates do not replace external validation.

CRP provided incremental discriminatory information within the present dataset; however, the absence of overlap between groups makes the apparent performance highly susceptible to spectrum and selection bias.

The model is exploratory and is not proposed as a stand-alone diagnostic tool.

**Figure 5:**
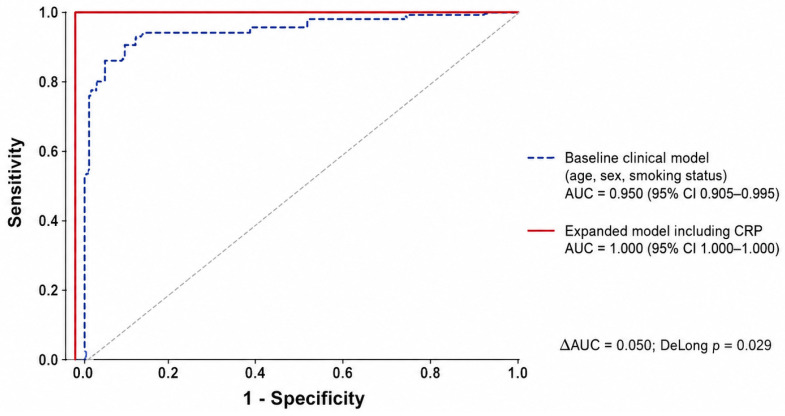
Comparative receiver operating characteristic curves for the baseline clinical model and the expanded model including CRP. Receiver operating characteristic curves comparing the baseline clinical model, including age, sex, and smoking status, with the expanded model additionally including C-reactive protein. The baseline model achieved an AUC of 0.950, whereas the expanded model achieved an apparent AUC of 1.000. The absolute difference in AUC was 0.050 (DeLong *p* = 0.029). The perfect apparent discrimination of the expanded model should be interpreted cautiously because CRP values did not overlap between the two diagnostic groups. AUC, area under the curve; CRP, C-reactive protein.

These results indicate that CRP provided incremental discriminatory information beyond age, sex, and smoking status in the present dataset. Nevertheless, CRP values showed no overlap between the diagnostic groups, and the study used a selected retrospective cohort with equal numbers of eligible NPC and AH patients. The estimates should therefore be interpreted as exploratory and require prospective external validation before clinical application (Figure 5).

## 4. Discussion

The present study explored the inflammatory–hematological profile of patients with nasopharyngeal carcinoma (NPC) compared with patients presenting adenoid hypertrophy and clinical suspicion of malignancy. The results demonstrate that routine inflammatory and hematological biomarkers may provide clinically relevant information for the differential diagnostic evaluation of nasopharyngeal lesions and for the characterization of systemic inflammatory responses.

One of the most important findings of the study was the markedly elevated systemic inflammatory response observed in the NPC group. Patients with nasopharyngeal carcinoma showed substantially higher CRP, ESR, and leukocyte values compared with patients diagnosed with benign adenoid hypertrophy. These findings indicate a more pronounced systemic inflammatory response in the NPC group. Previous NPC-specific studies have reported associations between inflammatory biomarkers, cytokine signaling, and the tumor microenvironment; however, these mechanisms were not directly investigated in the present study [20,21,22].

CRP demonstrated particularly strong discriminatory performance between malignant and benign disease. This observation is consistent with previous oncological studies reporting associations between elevated CRP concentrations and cancer presence, recurrence, treatment response, and unfavorable clinical outcomes. Similarly, elevated ESR values in the NPC group further support the presence of chronic inflammatory activation associated with malignant progression [23,24,25].

Interestingly, despite higher classical inflammatory markers in the NPC group, derived immune-inflammatory indices such as NLR, PLR, and SII demonstrated paradoxically higher values in the adenoid hypertrophy group [26,27,28]. PLR was higher in the AH group, whereas NLR and SII did not differ significantly between groups. These findings should be interpreted as exploratory between-group differences and may be influenced by age imbalance, cohort selection, residual confounding, or measurement-related factors.

The higher PLR observed in the AH group may reflect differences in the inflammatory response between benign lymphoid hyperplasia and malignant disease. However, because immune-cell function, cytokine profiles, and tissue immunology were not assessed, the biological mechanisms underlying these differences cannot be determined from the present study [29,30,31].

These findings suggest that composite inflammatory biomarkers may reflect different aspects of the systemic inflammatory response rather than inflammation intensity alone. Consequently, elevated NLR, PLR, and SII values should not automatically be interpreted as markers of malignancy, particularly in diseases characterized by chronic lymphoid hyperplasia and persistent mucosal immune stimulation [32,33,34].

Another important observation was the marked heterogeneity of inflammatory responses among NPC patients. Higher inflammatory marker levels observed in the NPC group may reflect the inflammatory response associated with malignant disease. However, because tumor extent and endoscopic characteristics were not systematically evaluated, no conclusions regarding tumor aggressiveness or disease extent can be drawn from the present study. This observation may have important clinical implications, as reliance exclusively on systemic inflammatory markers could underestimate disease severity in selected patients [35,36].

The variability in inflammatory marker concentrations among patients with NPC suggests that systemic inflammatory responses are not uniform across individuals [37]. Because standardized endoscopic assessment and tumor staging were not incorporated into the present analysis, the relationship between inflammatory biomarkers and local disease characteristics remains unknown [38,39].

The study also identified mild alterations in hepatic biochemical markers, particularly GGT and transaminases. Although these changes were not among the strongest discriminatory variables, they may suggest systemic metabolic and inflammatory involvement associated with chronic tumor-related stress. The interaction between systemic inflammation, oxidative stress, hepatic metabolism, and oncological disease progression remains insufficiently explored in nasopharyngeal carcinoma and may represent an important direction for future research [40,41,42].

From a clinical perspective, the present findings support the potential utility of low-cost routine biomarkers in the differential diagnostic evaluation of suspicious nasopharyngeal lesions. In many clinical settings, especially in resource-limited environments, access to advanced molecular or viral testing may be restricted. In this context, accessible inflammatory markers such as CRP, ESR, leukocyte count, and derived inflammatory indices may contribute to early risk stratification and prioritization of patients requiring extensive oncological evaluation [43].

Importantly, the study emphasizes that isolated biomarkers should not be interpreted independently. Instead, inflammatory biomarkers should be integrated with clinical examination, imaging findings, endoscopic appearance, and histopathological evaluation to improve diagnostic accuracy and avoid overinterpretation of isolated inflammatory abnormalities.

Overall, the findings highlight the complexity of systemic inflammatory responses in nasopharyngeal pathology and suggest that routinely available inflammatory biomarkers may complement the differential diagnostic evaluation of suspicious nasopharyngeal lesions. Further prospective studies integrating endoscopic findings, tumor staging, and immune profiling are needed to clarify the biological mechanisms underlying these observations.

### Study Limitations

Several limitations of the present study should be acknowledged. First, the study was conducted using a retrospective single-center design, which may limit the generalizability of the findings and restrict causal interpretation of the observed associations. Second, the sample size was relatively limited, particularly considering the inter-individual variability in inflammatory marker profiles, potentially reducing statistical power for subgroup analyses and exploratory clustering interpretation.

Third, although routine inflammatory and hematological biomarkers demonstrated important diagnostic and biological associations, advanced molecular and immunological characterization was not available. Epstein–Barr virus (EBV) viral load, cytokine profiling, tumor microenvironment markers, and genomic analyses were not included, limiting mechanistic interpretation of the observed inflammatory patterns.

Additionally, the derived inflammatory indices and the observed patterns of inflammatory biomarkers proposed in the present study were based on routinely available laboratory parameters and therefore require external validation in larger prospective cohorts. The proposed inflammatory marker patterns and inflammatory process should consequently be interpreted as exploratory biological concepts rather than fully validated clinical entities.

Another limitation involves the potential influence of confounding inflammatory factors that may not have been completely controlled despite the exclusion criteria, including subclinical infections, smoking-related inflammatory burden, metabolic status, and environmental exposures.

Furthermore, the study focused primarily on inflammatory–hematological and biochemical parameters without integrating advanced imaging quantification or longitudinal oncological outcomes such as progression-free survival, recurrence, or overall survival. Consequently, the prognostic implications of the observed inflammatory marker patterns remain incompletely defined.

Additionally, this study did not include standardized endoscopic tumor assessment, immune profiling, or cytokine measurements. Consequently, potential mechanistic explanations regarding tumor aggressiveness, immunological status, or inflammatory marker patterns should be considered hypothesis-generating rather than evidence directly supported by the present data.

Despite these limitations, the study provides an integrated clinicobiological perspective regarding inflammatory heterogeneity in nasopharyngeal pathology and highlights the potential utility of routine low-cost biomarkers in differentiating malignant and benign nasopharyngeal disease.

## 5. Conclusions

In this selected single-center cohort, CRP, ESR, and leukocyte count were higher in node-positive NPC than in suspicious histopathologically benign AH, while PLR was higher in AH and NLR and SII did not differ significantly.

CRP and leukocyte count showed apparent complete discrimination, but this result does not establish stand-alone diagnostic utility because of the selected groups, age im-balance, small sample, and absence of external validation. Prospective studies in consecutive patients with standardized laboratory, EBV-related, imaging, and histopathological assessment are required.

The findings do not establish causality, disease-stage associations, prognostic value, treatment-response prediction, or clinical utility as a stand-alone diagnostic test. The substantial age imbalance, selected comparator group, lack of EBV-related variables, and absence of external validation limit generalizability. Prospective studies involving consecutive patients, standardized laboratory measurements, EBV-related testing, and an independent validation cohort are required.

## Figures and Tables

**Table 1:** Demographic, hematological, inflammatory, and biochemical characteristics of the study population.

Variable	Mean ± SD	Range
Age (years)	47.10 ± 12.00	24–71
Male sex, *n* (%)	63 (87.5%)	—
Smoking, *n* (%)	64 (88.9%)	—
Alcohol consumption, *n* (%)	68 (94.4%)	—
Leukocyte count (×10^3^/µL), *n* = 69	13.50 ± 5.76	4.91–25.00
Erythrocytes (×10^6^/µL)	5.34 ± 0.73	3.99–6.51
Hemoglobin (g/dL)	14.47 ± 2.05	11.50–18.90
Hematocrit (%)	44.38 ± 5.46	38.22–50.24
Platelet count (×10^3^/µL)	263.10 ± 45.26	195.07–345.03
Neutrophils (%)	70.35 ± 8.21	45.70–80.72
Lymphocytes (%)	34.31 ± 17.57	19.40–61.22
Monocytes (%)	11.43 ± 3.33	5.25–17.52
CRP (mg/L)	42.71 ± 42.40	0.75–105.00
ESR (mm/h)	34.19 ± 45.58	3–150
Glucose (mg/dL)	105.97 ± 30.46	70–167
Urea (mg/dL)	34.01 ± 5.89	17.83–42.20
Creatinine (mg/dL)	0.82 ± 0.27	0.43–1.76
AST (U/L)	26.72 ± 5.90	22.50–42.10
ALT (U/L)	26.85 ± 7.47	22.10–45.17
GGT (U/L)	40.14 ± 13.45	30.35–71.27
Triglycerides (mg/dL)	78.27 ± 32.24	59.05–160.97
INR	1.16 ± 0.15	0.75–1.40
APTT (s)	26.78 ± 4.32	22.18–34.21
NLR	1.80 ± 0.72	0.38–3.53
PLR	78.06 ± 23.90	38.66–118.98
SII (×10^3^/µL)	454.36 ± 168.62	114.82–782.61

Abbreviations: APTT, activated partial thromboplastin time; AST, aspartate aminotransferase; ALT, alanine aminotransferase; CRP, C-reactive protein; ESR, erythrocyte sedimentation rate; GGT, gamma-glutamyl transferase; INR, international normalized ratio; NLR, neutrophil-to-lymphocyte ratio; PLR, platelet-to-lymphocyte ratio; SII, systemic immune-inflammation index. Three clinically implausible leukocyte-count values—0.00, 0.10, and 0.12 × 10^3^/µL—were treated as missing; therefore, leukocyte-count statistics were calculated for 69 patients. NLR, PLR, and SII were recalculated using absolute neutrophil, absolute lymphocyte, and platelet counts obtained from the same complete blood count. Three clinically implausible leukocyte-count values—0.00, 0.10, and 0.12 × 10^3^/µL—were treated as missing. Therefore, the descriptive leukocyte-count statistics were calculated using 69 evaluable patients.

**Table 2:** Group-specific baseline, inflammatory, and hematological characteristics of patients with nasopharyngeal carcinoma and suspicious histopathologically benign adenoid hypertrophy.

Variable	NPC Group (*n* = 36)	AH Group (*n* = 36)	*p*-Value
Age (years)	38.14 ± 7.64	56.06 ± 8.29	<0.001
Male sex, *n* (%)	32 (88.9%)	31 (86.1%)	1.000
Smoking, *n* (%)	32 (88.9%)	32 (88.9%)	1.000
Alcohol consumption, *n* (%)	34 (94.4%)	34 (94.4%)	1.000
Urban residence, *n* (%)	28 (77.8%)	29 (80.6%)	1.000
Laterocervical lymph-node involvement, *n* (%)	36 (100.0%)	0 (0.0%)	<0.001
Leukocyte count (×10^3^/µL)	17.43 (15.06–20.15)	8.50 (8.16–9.50)	<0.001
Absolute neutrophil count (×10^3^/µL)	6.58 (5.68–6.85)	6.29 (5.05–6.71)	1.000
Absolute lymphocyte count (×10^3^/µL)	4.08 (3.06–5.80)	2.90 (2.75–3.07)	<0.001
Platelet count (×10^3^/µL)	262.76 (225.01–301.64)	245.02 (224.27–307.92)	0.545
CRP (mg/L)	79.50 (72.50–100.50)	2.08 (1.27–3.36)	<0.001
ESR (mm/h)	30.50 (15.75–71.25)	12.00 (8.00–14.00)	<0.001
AST (U/L)	24.51 (23.77–24.92)	24.85 (23.80–32.80)	0.287
ALT (U/L)	23.20 (22.70–28.92)	25.17 (22.88–25.20)	0.448
GGT (U/L)	32.75 (31.96–42.57)	33.44 (32.59–45.38)	0.171
NLR	1.82 (1.22–2.15)	2.11 (1.59–2.22)	0.188
PLR	66.23 (53.86–73.59)	98.75 (73.24–112.73)	<0.001
SII (×10^3^/µL)	467.08 (365.89–484.79)	482.94 (444.70–711.48)	0.128

Data are presented as mean ± standard deviation for age, number and percentage for categorical variables, and median (interquartile range) for laboratory variables. Age was compared using Welch’s *t*-test, categorical variables using Fisher’s exact test, and laboratory variables using the Mann–Whitney U test. NLR, PLR, and SII were recalculated using absolute neutrophil, absolute lymphocyte, and platelet counts obtained from the same pretreatment complete blood count. Three clinically implausible leukocyte-count values (0.00, 0.10, and 0.12 × 10^3^/µL), all belonging to the AH group, were treated as missing; therefore, leukocyte-count comparisons included 36 patients with NPC and 33 patients with AH. NPC, nasopharyngeal carcinoma; AH, adenoid hypertrophy; CRP, C-reactive protein; ESR, erythrocyte sedimentation rate; AST, aspartate aminotransferase; ALT, alanine aminotransferase; GGT, gamma-glutamyl transferase; NLR, neutrophil-to-lymphocyte ratio; PLR, platelet-to-lymphocyte ratio; SII, systemic immune-inflammation index.

**Table 3:** Exploratory diagnostic performance of routine inflammatory biomarkers for differentiating nasopharyngeal carcinoma from adenoid hypertrophy.

Biomarker	Direction Indicating NPC	AUC	95% CI	*p*	Optimal Cut-Off (Youden)	Sensitivity (%)	Specificity (%)
CRP	Higher	1.000	1.000–1.000	<0.001	>50.00	100.0	100.0
ESR	Higher	0.948	0.889–0.988	<0.001	>15.00	100.0	83.3
Leukocyte count (×10^3^/µL)	Higher	1.000	1.000–1.000	<0.001	>10.97	100.0	100.0
NLR	Lower	0.590	0.449–0.727	0.188	≤1.50	50.0	86.1
PLR	Lower	0.833	0.725–0.915	<0.001	≤73.77	100.0	66.7
SII	Lower	0.604	0.466–0.731	0.128	≤485.41	100.0	36.1
GGT	Higher	0.406	0.262–0.546	0.171	—	—	—

AUC, area under the receiver operating characteristic curve; CI, confidence interval; CRP, C-reactive protein; ESR, erythrocyte sedimentation rate; GGT, gamma-glutamyl transferase; NLR, neutrophil-to-lymphocyte ratio; PLR, platelet-to-lymphocyte ratio; SII, systemic immune-inflammation index. NPC was coded as the positive outcome. Cut-off values were selected exploratorily by maximizing the Youden index. Because of the selected study population and the absence of external validation, the reported AUCs should be interpreted as exploratory rather than definitive estimates of diagnostic accuracy.

**Table 4:** Comparative performance of the baseline clinical and CRP-expanded diagnostic models.

Performance Measure	Baseline Clinical Model	Expanded Model Including CRP
AUC (95% CI)	0.950 (0.905–0.995)	1.000 (1.000–1.000)
Sensitivity, %	86.1	100.0
Specificity, %	88.9	100.0
Brier score	0.0905	0.0024
Calibration intercept	0.012	−0.490
Calibration slope	1.049	1.260
Optimism-corrected AUC	0.938	1.000
Optimism-corrected Brier score	0.1073	0.0036

The baseline clinical model included age, sex, and smoking status. The expanded model additionally included CRP per 10 mg/L increase.

## Data Availability

The original contributions presented in this study are included in this article. Statistical code available in Supplementary File S1.

## Supplementary Materials

The following supporting information can be downloaded at https://www.mdpi.com/article/10.3390/diagnostics16152469/s1. Table S1. Complete coefficient output for the baseline Firth penalized logistic regression model; Table S2. Complete coefficient output for the CRP-expanded Firth penalized logistic regression model; Table S3. Apparent and bootstrap-corrected performance of the baseline and CRP-expanded Firth logistic regression models; Table S4. Complete statistical comparison of the baseline clinical and CRP-expanded Firth logistic regression models; File S1. Fully reproducible Python code used for patient-level data auditing, recalculation of derived inflammatory indices, descriptive and comparative analyses, Spearman correlation analysis, ROC analysis, Firth penalized logistic regression, model comparison, and stratified bootstrap internal validation.

## Abbreviations

The following abbreviations are used in this manuscript:

Abbreviation	Definition
NPC	Nasopharyngeal carcinoma
AH	Adenoid hypertrophy
CRP	C-reactive protein
ESR	Erythrocyte sedimentation rate
NLR	Neutrophil-to-lymphocyte ratio
PLR	Platelet-to-lymphocyte ratio
SII	Systemic immune-inflammation index
GGT	Gamma-glutamyl transferase
AST	Aspartate aminotransferase
ALT	Alanine aminotransferase
INR	International normalized ratio
APTT	Activated partial thromboplastin time
ROC	Receiver operating characteristic
AUC	Area under the curve
CI	Confidence interval
SD	Standard deviation
EBV	Epstein–Barr virus
ENT	Ear, nose, and throat
SPSS	Statistical Package for the Social Sciences
