# Supplementary material for: Inflammatory–Hematological Profiles in Nasopharyngeal Carcinoma and Suspicious Adenoid Hypertrophy: An Exploratory Single-Center Study
Source: Diagnostics (Basel). 2026 Aug 5;16(15):2469. doi: 10.3390/diagnostics16152469 (PMC13465784; doi:10.3390/diagnostics16152469)
Supplement: Supplementary file 1 [file diagnostics-16-02469-s001.zip › diagnostics-4423841-Supplementary File S1.pdf]

File S1: Fully reproducible Python code used for patient-level data auditing, recalculation of derived inflammatory indices, descriptive and comparative analyses, Spearman correlation analysis, ROC analysis, Firth penalized logistic regression, model comparison, and stratified bootstrap internal validation. Python >= 3.11:

- numpy >= 2.0
- pandas >= 2.2
- scipy >= 1.13
- scikit-learn >= 1.5
- matplotlib >= 3.9
- openpyxl >= 3.1
