# Supplementary material for: Inflammatory–Hematological Profiles in Nasopharyngeal Carcinoma and Suspicious Adenoid Hypertrophy: An Exploratory Single-Center Study
Source: Diagnostics (Basel). 2026 Aug 5;16(15):2469. doi: 10.3390/diagnostics16152469 (PMC13465784; doi:10.3390/diagnostics16152469)
Supplement: Supplementary file 1 [file diagnostics-16-02469-s001.zip › diagnostics-4423841-Supplementary Tables.pdf]

Table S1. Complete coefficient output for the baseline Firth penalized logistic regression model

Predictor	β	SE	Adjusted OR	95% CI	Penalized LR χ^2	p-value
Intercept	13.196	3.443	538,372.48	1,588.51–2.97 × 10^9	32.062	<0.001
Age, per 1-year increase	−0.289	0.072	0.749	0.623–0.845	53.400	<0.001
Male sex, yes vs. no	0.034	0.943	1.035	0.173–6.670	0.001	0.970
Smoking, yes vs. no	0.466	1.301	1.594	0.102–20.540	0.132	0.716

Histopathologically confirmed nasopharyngeal carcinoma was coded as 1 and histopathologically benign adenoid hypertrophy as 0. The model included age, sex, and smoking status. Sex was recoded as 1 for male and 0 for female, while smoking was coded as 1 for yes and 0 for no. Adjusted odds ratios were obtained by exponentiating the Firth regression coefficients. Confidence intervals represent profile penalized-likelihood 95% confidence intervals, and p-values were calculated using penalized likelihood-ratio tests. The analysis included 72 patients: 36 with NPC and 36 with AH.

Table S2. Complete coefficient output for the CRP-expanded Firth penalized logistic regression model

Predictor	β	SE	Adjusted OR	95% CI	Penalized LR χ^2	p-value
Intercept	−1.357	7.753	0.257	3.83 × 10^{-13}–8.61 × 10^7	0.033	0.855
Age, per 1-year increase	0.002	0.130	1.002	0.674–1.450	<0.001	0.987
Male sex, yes vs. no	−1.206	2.177	0.299	0.002–27,297.025	0.245	0.620
Smoking, yes vs. no	−1.424	2.207	0.241	0.001–5200.355	0.254	0.614
CRP, per 10 mg/L increase	1.152	0.469	3.164	1.679–16.720	30.984	<0.001

Histopathologically confirmed nasopharyngeal carcinoma was coded as 1 and histopathologically benign adenoid hypertrophy as 0. The expanded model included age, sex, smoking status, and C-reactive protein expressed per 10 mg/L increase. Sex was coded as 1 for male and 0 for female, while smoking was coded as 1 for yes and 0 for no. Adjusted odds ratios were obtained by exponentiating the Firth regression coefficients. Confidence intervals represent profile penalized-likelihood 95% confidence intervals, and p-values were calculated using penalized likelihood-ratio tests. The analysis included 72 patients: 36 with NPC and 36 with AH.

Table S3. Apparent and bootstrap-corrected performance of the baseline and CRP-expanded Firth logistic regression models

Performance measure	Baseline clinical model	Expanded model including CRP
Apparent AUC	0.950	1.000
Sensitivity at threshold 0.50, %	86.1	100.0
Specificity at threshold 0.50, %	88.9	100.0
Apparent Brier score	0.0905	0.0024
Calibration intercept	0.010	−0.437

Calibration slope	1.049	1.260
Optimism-corrected AUC	0.938	1.000
Optimism-corrected Brier score	0.1067	0.0036
Successful bootstrap resamples, n	1000	1000
Failed bootstrap iterations, n	0	0

Histopathologically confirmed nasopharyngeal carcinoma was coded as 1 and histopathologically benign adenoid hypertrophy as 0. The baseline clinical model included age, sex, and smoking status, whereas the expanded model additionally included C-reactive protein per 10 mg/L increase. Sensitivity and specificity were calculated at a predicted-probability threshold of 0.50. Internal validation was performed using 1000 stratified bootstrap resamples. Optimism-corrected performance estimates were obtained by correcting the apparent AUC and Brier score for mean bootstrap optimism. Calibration intercept and slope were estimated by Firth logistic recalibration of the observed outcome on the model linear predictor.

Table S4. Complete statistical comparison of the baseline clinical and CRP-expanded Firth logistic regression models

Comparison measure	Baseline model	Expanded model	Difference/statistic	df	p-value
Apparent AUC	0.950	1.000	ΔAUC = 0.050	—	—
DeLong comparison of correlated AUCs	0.950	1.000	z = 2.178	—	0.029
Penalized log-likelihood	−17.165	0.474	Difference = 17.640	—	—
Penalized likelihood-ratio test	—	—	χ^2 = 35.279	1	<0.001

Histopathologically confirmed nasopharyngeal carcinoma was coded as 1 and histopathologically benign adenoid hypertrophy as 0. The baseline model included age, sex, and smoking status, whereas the expanded model additionally included CRP per 10 mg/L increase. The apparent AUC increased from 0.950 to 1.000, corresponding to an absolute increase of 0.050. Correlated AUCs were compared using the DeLong test. Nested models were additionally compared using a penalized likelihood-ratio test. The analysis included 72 patients: 36 with NPC and 36 with AH.
